# Lifecourse Activity Participation From Early, Mid, and Later Adulthood as Determinants of Cognitive Aging: The Lothian Birth Cohort 1921

**DOI:** 10.1093/geronb/gbw124

**Published:** 2016-10-07

**Authors:** Alan J. Gow, Alison Pattie, Ian J. Deary

**Affiliations:** ^1^Department of Psychology, Heriot-Watt University, Edinburgh.; ^2^Centre for Cognitive Ageing and Cognitive Epidemiology and; ^3^Department of Psychology, School of Philosophy, Psychology and Language Sciences, University of Edinburgh.

**Keywords:** Differential preservation, Leisure activity, Longitudinal, Physical activity, Preserved differentiation, Retrospective

## Abstract

**Objectives:**

To examine potential sensitive periods for activity participation across adulthood to reduce cognitive decline and to determine whether associations persist after accounting for the lifetime stability of cognitive ability.

**Method:**

The Lothian Birth Cohort 1921 is a longitudinal study of cognitive aging. Participants were born in 1921 and most completed a mental ability test at the age of 11 years. Cognitive assessments were completed at mean ages 79 (*N* = 550), 83 (*N* = 321), 87 (*N* = 235), and 90 years (*N* = 129). Participants provided retrospective details of their activity participation for young (20–35 years), mid (40–55 years), and later adulthood (60–75 years), and contemporaneously at age 79.

**Results:**

Associations between activity and the level of, and change in, cognitive ability in old age were examined with latent growth curve models. Accounting for demographics and childhood cognitive ability, engagement in leisure activities in midlife was positively associated with cognitive ability level (path coefficient = .32), whereas higher physical activity in later adulthood was associated with less cognitive decline (.27).

**Discussion:**

The findings support a lifecourse approach in identifying determinants of cognitive aging; leisure and physical activity during different periods of adulthood may enhance cognitive abilities or reduce decline.

Understanding the determinants of cognitive aging is a research priority, and although a number of potentially neuroprotective factors have been identified (Hertzog, Kramer, Wilson, & Lindenberger, 2009), lifecourse considerations need to be addressed to better understand what protects (or harms) cognitive abilities with age. Lifestyle and behavioral factors are of particular interest, given that these might be amenable to intervention (Anstey & Christensen, 2000). Whereas activity participation across intellectual, social, and physical domains has been reported as advantageous in terms of maintained cognitive ability with age or slower decline (Fratiglioni, Wang, Ericsson, Maytan, & Winblad, 2000; Hertzog et al., 2009; Lyketsos, 2006), the current study sought to address two major concerns in exploring these associations. Firstly, associations between cognitive aging and activity participation, particularly in the socio-intellectual domain, are prone to confounding by reverse causation (Bielak, 2010; Bielak, Anstey, Christensen, & Windsor, 2012; Gow, Avlund, & Mortensen, 2014; Gow, Corley, Starr, & Deary, 2012; Salthouse, 2006); as the current cohort completed a test of cognitive ability in childhood, the associations between lifetime activity and cognitive aging were examined before and after accounting for prior cognitive ability. Secondly, few studies have an extended follow-up throughout midlife and into older age (Andel, Silverstein, & Kåreholt, 2015). One alternative is to utilize retrospective assessment methods within existing aging cohorts (Wilson, Barnes, & Bennett, 2003). Using this approach, the current study examined whether there might be sensitive periods for activity participation across adulthood that are cognitively beneficial in old age.

It has been said that “on balance, the available evidence favors the hypothesis that maintaining an intellectually engaged and physically active lifestyle promotes successful cognitive aging” (Hertzog et al., 2009, p. 1). The hypothesis is intuitively acceptable given the expectation that individuals “who engage in activities that make significant demands on their cognitive skills will show greater maintenance or improvement in their abilities” (Hultsch, Hertzog, Small, & Dixon, 1999, p. 247).

Remaining mentally engaged is often seen as the most important activity with respect to the preservation of cognitive ability into old age, frequently expressed as “use it or lose it” (Kramer, Bherer, Colcombe, Dong, & Greenough, 2004). Although the number of studies reporting positive associations between socio-intellectual or physical activity cannot be questioned (Fratiglioni, Paillard-Borg, & Winblad, 2004; Hertzog et al., 2009), Bielak (2010) posed at least seven questions which need to be addressed before a “use it or lose it” model of engagement and cognitive aging should be accepted. Two of these questions motivated the current study: “Does activity participation impact cognition, or does cognition impact activity participation?” and “How long does past activity impact current cognition?” (Bielak, 2010).

## Does Activity Participation Impact Cognition, or Does Cognition Impact Activity Participation?

In the majority of studies, results have been adjusted for age, sex, and education, in addition to baseline cognitive ability on average about 6–7 years prior to a follow-up examination (Fratiglioni et al., 2004). Even if associations remain between activity and cognitive ability after adjustment for these potential confounders, the nature of causality is difficult to tease out, more so as participation in cognitively demanding activities is likely to be at least partially determined by prior cognitive ability (Hultsch et al., 1999). For example, in a sample related to the current one, the Lothian Birth Cohort 1936 for whom childhood cognitive ability data were also available, leisure activity participation at age 70 was associated with contemporaneously assessed cognitive ability. However, this was completely attenuated when childhood cognitive ability was included; the association between physical activity and cognitive ability was unaffected by this adjustment (Gow et al., 2012). That is not to say that prior ability would always account for later activity-cognitive ability associations.

The National Survey of Health and Development (NSHD) began with a sample of more than 5,000 children from England, Scotland, and Wales born in March 1946 (Wadsworth, 1991), and importantly, included tests of cognitive ability during childhood. Assessments of physical and spare-time leisure activity participation were conducted at ages 36 and 43; memory was also assessed on these occasions, and again at age 53 (Richards, Hardy, & Wadsworth, 2003). The results suggested that, independent of childhood ability, active leisure time was associated with the level of memory function, whereas physical activity was associated with reduced decline over 10 years. The analysis further suggested that being physically active at 36 years of age offered minimal protection against decline if participants were physically inactive at 43 years of age. Physical activity appeared to offer a cumulative beneficial effect, “with those engaged at both occasions having an average decline 1.01 points slower than those engaged at neither age” (Richards et al., 2003, p. 790). By being able to adjust for childhood ability, the results were less likely to be a consequence of reverse causality—that is, those of higher prior cognitive ability being more active. And yet, the relatively young age of the cohort does highlight an important issue for other researchers in the field. Most studies begin with individuals when aged about 60, 70, or older; however, by their 50s, the NSHD participants had already experienced cognitive changes apparently associated with their activity participation. In another study harnessing archival information (in this case high school yearbooks for activity data and cognitive ability tests administered at school), both childhood IQ and activity level predicted dementia/mild cognitive impairment status (Fritsch et al., 2005). Although a crude measure of activity, it perhaps has advantages over other retrospective assessments in not being confounded by participant biases in recall (though only pursuits within school were listed).

Whereas these studies suggest benefits of activity participation independently of prior ability, it is noted these either considered cognitive status at a single time point, or cognitive change through midlife (rather than old age). Studies have highlighted that associations with cognitive ability level in old age may well exist in the absence of any associations with subsequent cognitive change (Bielak et al., 2012; Gow et al., 2014; Richards et al., 2003). Although studies tracking both cognitive ability and activity change within old age have addressed the causality issue with the use of dual-change approaches for example, and appear to favor activity participation affecting cognitive change (Ghisletta, Bickel, & Lövdén, 2006; Lovden, Ghisletta, & Lindenberger, 2005), it is unclear how the lifetime stability of cognitive ability might affect these conclusions.

Thus, a critical issue in cognitive aging research remains in distinguishing differential preservation from preserved differentiation (Salthouse, 2006):

The key difference between these two perspectives is that the differential-preservation hypothesis views mental activity as a factor that protects against age-related decline in mental ability, whereas the preserved-differentiation hypothesis views an individual’s current level of mental activity as at least partly a manifestation of his or her prior level of mental ability (p. 70).

The current work is therefore primarily driven by this need to better understand activity-cognitive ability associations.

## How Long Does Past Activity Impact Current Cognition?

Many longitudinal studies of cognitive aging follow a similar pattern: Baseline examination is conducted when participants have entered late adulthood, including cognitive and activity assessments; cognitive follow-up is performed some years later (often between 2 and 10 years); and this allows an investigation of whether cognitive change over the study period is predicted by baseline activity levels (Hertzog et al., 2009). Although some studies have tracked both activity and cognitive change over the same period (Bielak et al., 2012; Mitchell et al., 2012), most rely on assessments of activity in later life, and any cognitive changes from that point onwards (Tomioka, Kurumatani, & Hosoi, in press; Wilson, Bennett, et al., 2003). This makes it more difficult to determine whether it is the level of activity in later life which is important, or whether lifetime activity participation may be crucial. Lifelong activity participation may be an indicator of the use of an individual’s cognitive abilities in their day-to-day living (Andel et al., 2015; Friedland et al., 2001; Wilson, Barnes, et al., 2003).

To address this issue, it would be necessary for studies to assess activity and cognition in mid-adulthood, for example, and then follow the participants over an extended period of time into old age. Studies of this nature are rare, though many midlife cohorts (Richards et al., 2003) are of increasing interest to the cognitive aging community as they enter old age. A second option would be to assess activity participation retrospectively from some baseline examination and determine whether the lifetime level of, or change in, activity is associated with cognitive ability level and subsequent change (Wilson, Barnes, et al., 2003).

In a case–control design, Friedland and colleagues (2001) suggested that those who performed fewer activities in early or mid-adulthood (aged 20–39 and 40–59 years, respectively) had greater odds of developing Alzheimer’s disease and, interestingly, that the probability was significantly greater for those showing a reduction in intellectual activity participation between early and mid-adulthood. Although innovative, the activity data were provided by “significant others” rather than the participants themselves which may confound the reported associations.

Wilson and colleagues (2003), interested in whether cumulative exposure to more intellectual stimulation might influence cognitive ability in later life, constructed a retrospective measure of lifetime participation in cognitive activities. Twenty-five items were chosen to represent common cognitive activities with reduced physical or social components (such as “visit library” or “write letter”), split over five ages: 6, 12, 18, 40, and 84 years, the latter being the time of the examination. Three of the five domains of cognitive ability assessed were associated with lifetime cognitive activity (perceptual speed, visuospatial ability, and semantic memory, but not episodic or working memory) after adjustment for age and sex, and continued to be so when education was also included (Wilson, Barnes et al., 2003). The conclusion might therefore be that higher lifetime cognitive activity predicts better cognitive functioning in later life, and this conclusion would be more persuasive still if the association continues to hold after adjustment for the level of prior cognitive ability.

## The Current Study

Retrospective assessments of activity were completed within the Lothian Birth Cohort 1921 (LBC1921), an ongoing study of cognitive aging. Three distinct age periods were considered (20–35, 40–55, and 60–75 years) to examine whether there might exist sensitive periods for activity participation and to investigate whether such associations might differ by leisure and physical activity. As the cohort also has cognitive ability assessments from childhood, the key aim of the analysis was to explore whether any activity-cognitive ability associations were confounded by differential preservation; that is, do the associations persist when the lifetime stability of cognitive ability is accounted for?

## Method

### Participants

The Lothian Birth Cohort 1921 (LBC1921) participants were born in 1921, and most had participated in the Scottish Mental Survey 1932 when aged 11 years (a national survey of mental ability) (Scottish Council for Research in Education, 1933). They were recruited into the LBC1921 at mean age of 79 years (*N* = 550; Wave 1) and completed detailed cognitive, psychosocial, medical, and physical assessments. These were repeated on four further occasions: at mean ages 83 (*N* = 321; Wave 2), 87 (*N* = 235; Wave 3), 90 (*N* = 129; Wave 4), and 92 (*N* = 59; Wave 5) (Deary, Whalley, & Starr, 2009; Deary, Whiteman, Starr, Whalley, & Fox, 2004; Gow et al., 2011). As the sample size was much reduced at the age-92 assessment, the current analyses focus on data collected from ages 79 to 90 years (Waves 1–4).

During the second wave of assessment (age 83), participants were asked to complete a retrospective lifestyle questionnaire to assess work history, lifetime social support, lifetime activity participation, and religiosity/spirituality. All 488 participants listed in the LBC1921 at that time were mailed a questionnaire booklet (this is higher than the final number tested at Wave 2 as some participants had incomplete data from Wave 1 and were then not invited to return for Wave 2, while others later chose not to return). All returned questionnaires were checked for omissions, multiple responses, and incongruent answers. If any of these were found, they were detailed in a letter sent to the participants asking for corrections. With reminders and correction letters, the final response was as follows: of the 488 participants mailed the questionnaire booklet, 444 (91.0%) responded, comprising questionnaires from 384 participants (78.7% of those mailed), 59 (12.1% of those mailed) who refused to complete the questionnaires, and one participant who could not be linked in the database. At the end of the data collection period, 323 questionnaire booklets (84.1% of those returned) were fully complete and 61 (15.9%) remained partially completed after corrections were requested, where appropriate. Sample sizes for the activity questionnaires are detailed below.

### Measures

#### Cognitive tests

Participants completed a battery of tests at each wave; the three tests completed across all waves were considered in the current analyses, being Verbal Fluency, Logical Memory, and Raven’s Standard Progressive Matrices.

Phonemic Verbal Fluency (Lezak, 1995) assessed an aspect of executive function, with participants asked to name as many words beginning with a target letter within 1 minute. Participants completed the task three times with different letters (C, F, and L), and the score was the number of words produced. Logical Memory from the Wechsler Memory Scale–Revised (Wechsler, 1987) was used to assess verbal declarative memory. Participants were read two short stories, and after each, their immediate recall was assessed. After a delay of about 25 minutes, participants were again required to recall the stories. A maximum score of 25 was available from each story, giving a total score of 100 across the immediate and delayed recall of the two stories. Raven’s Standard Progressive Matrices (Raven, Court, & Raven, 1977) assessed nonverbal fluid ability, requiring participants to choose a response (from a choice of 6 or 8) to complete a larger pattern. Participants were given a 20-minute time limit, and their score was the number of correct responses.

Participants had previously completed the Moray House Test No. 12 (MHT) at the age of 11 years (Scottish Council for Research in Education, 1933). The MHT is an omnibus test of mental ability, consisting of a range of items (e.g., analogies, arithmetic, and problem solving) with a maximum score of 76. The MHT has been validated against standardized cognitive ability tests in both childhood and later life (Deary et al., 2004).

#### Retrospective activity

In designing the retrospective questionnaire, there existed a period from age 11 to age 79 that was open for exploration. It was decided that the age periods included should be of equal duration and early, mid, and later adulthood were proposed as 20–35, 40–55, and 60–75 years, respectively. These periods were not driven by a specific theoretical perspective, but rather by the expectation of where the cohort were likely to be in terms of their lives: Early adulthood for most would likely be marked by entry and initial progression through their chosen career and starting a family; midlife by general stability in those key areas; and later adulthood by transitions to retirement (though these assumptions are returned to in the Discussion).

Participants provided details of their lifetime activity participation in the retrospective questionnaire booklet completed at age 83 (Wave 2). They were first asked to rate their general level of physical activity for three age periods, young (20–35 years old), mid (40–55 years old), and later adulthood (60–75 years old), on a 6-point scale: moving only in connection with necessary (household) chores; walking or other outdoor activities 1–2 times per week; walking or other outdoor activities several times per week; exercising 1–2 times per week to the point of perspiring and heavy breathing; exercising several times per week to the point of perspiring and heavy breathing; keep-fit/heavy exercise or competitive sport several times per week (Hirvensalo, Lintunen, & Rantanen, 2000).

Participants rated their frequency of participation in 15 socio-intellectual activities for the same age periods, drawn from those most commonly used in previous work (Glass, de Leon, Marottoli, & Berkman, 1999; Hultsch et al., 1999; Richards et al., 2003; Wilson, Barnes, et al., 2003; Wilson, Bennett, et al., 2003). The activities included, for example, visits to the library, reading a newspaper or magazine, and visits to friends or family. Participants were required to state on a 5-point scale (from every day or about every day to less than once a year/never) the frequency with which they generally did each activity during the specified age period. Exploratory factor analysis (EFA) at each age period defined leisure activity scores for young, mid, and later adulthood, detailed in Results (Retrospective Leisure Activity).

#### Age-79 activity

Physical and leisure activities were also assessed contemporaneously at age 79 (Wave 1). The 14 items from Glass and colleagues (1999) guided the development of an appropriate scale for leisure activity assessment, though items referring to preparing meals and shopping were removed from the list, because information about this had already been obtained from participants, and four items with a physical component were excluded. An item concerning “other paid employment” was also removed as it was felt this would not be relevant to the cohort (although “paid community work” was retained). Four items were added concerning talking to and visiting friends and relatives. In total, the activity questionnaire contained 13 items, and participants were required to select whether they never, rarely, sometimes, or frequently took part in each activity. As above, EFA was used to define a leisure activity score at the age of 79 years.

Participants were also asked on how many days in an average month they did sport or physical exercise (e.g., dancing or brisk walking) that made them out of breath and caused them to sweat, which they did for more than 20 minutes at a time.

#### Covariates

The following covariates collected at age 79 (Wave 1) were included in the analyses given their association with either activity and/or cognitive ability: sex, number of years in full-time education, social class (coded on a 5-point scale based on the highest occupational status of the household) (General Register Office, 1956), smoking (current, ex, and never) and estimated weekly alcohol consumption (in units).

#### Statistical analysis

**Figure 1.**
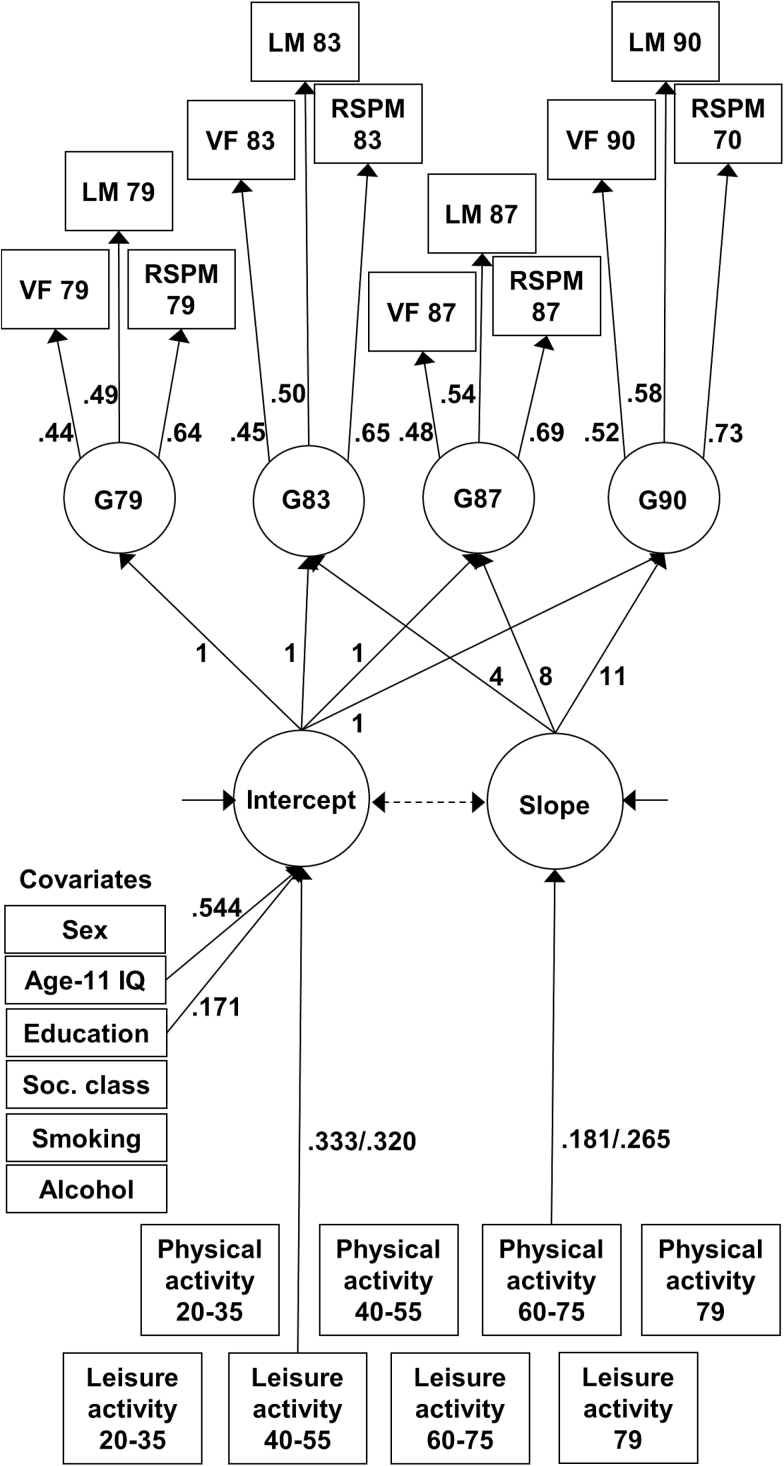
Latent growth curve model of the level of, and change in, general cognitive ability over four waves of the Lothian Birth Cohort 1921. In the latent growth curve model, manifest (measured) variables are represented by rectangles and latent traits by circles. Latent general cognitive ability factors (G) were produced at each occasion from the three cognitive tests completed (VF = Verbal Fluency, LM = Logical Memory, and RSPM = Raven’s Standard Progressive Matrices). Correlations between the activity factors are given in Supplementary Table 2 and between the cognitive ability variables in Supplementary Table 4. The principal outcome variables in the model are intercept (the level of general cognitive ability) and slope (the change in general cognitive ability across time). In a growth curve model with more than two occasions, the intercept is a composite representing overall level; here, the intercept term therefore represents the composite level of cognitive ability across ages 79 (Wave 1) to 90 (Wave 4). The measured variables have fixed contributions to the intercept; the fixed contributions to slope (4, 8, and 11) represent the number of years since the initial testing occasion, age 79 in this model. Figures with decimal points are the standardized estimates generated by the model, given before/after inclusion of the covariates. Only significant paths are shown (full results in Table 2), except the path from physical activity 60–75 to slope which was not significant before inclusion of the covariates.

Latent growth curve models were implemented in Mplus Version 5.2 to examine associations between activity variables and the level of, and change in, cognitive ability in old age (McArdle, 1986; Meredith & Tisak, 1990). Prior to analyses, cognitive ability scores were corrected for age in days at the time of testing, and all continuous variables were standardized. In the models, a latent general cognitive ability factor (g) was produced at ages 79 to 90 comprising Verbal Fluency, Logical Memory, and Raven’s Matrices (Figure 1). The fixed contributions to slope were considered as the number of years since the initial testing occasion at Wave 1 (age 79): 4 years to Wave 2, 8 years to Wave 3, and 11 years to Wave 4. Measurement invariance across the latent cognitive ability factors was tested using a four-stage procedure: the first model was run with all parameters varying freely; factor loadings were constrained equal in the next model; residual variances were then constrained; and, in a final model, the intercepts were also constrained. A deterioration in model fit at any stage would represent a failure of measurement invariance, with consequences for the interpretation of the slope parameter (Meredith, 1993).

Physical and leisure activity scores from the three retrospectively assessed periods (young, mid, and later adulthood) and at age 79 were included to assess their associations with the intercept (level of cognitive ability) and slope (change in cognitive ability) parameters, illustrated in Figure 1. Fully adjusted models were additionally run including the listed covariates and age-11 cognitive ability. Participants with data at baseline were included even if they were missing data at later waves using full information maximum likelihood, under the assumption this missingness was at random (Arbuckle, 1996).

## Results

Descriptive statistics for the covariates and cognitive ability are given in Table 1. As the modeling undertaken included all available cognitive ability data, the table displays descriptive statistics for the whole sample at any given wave and also for those who did and did not return at subsequent waves of testing.

**Table 1.** The Lothian Birth Cohort 1921: Descriptives From Age 79 to 90

	Age 79 (maximum *N* = 550)	Age 83 returnees (maximum *N* = 321)	Age 83 nonreturnees (maximum *N* = 229)	Age 87 returnees (maximum *N* = 235)	Age 87 nonreturnees (maximum *N* = 315)	Age 90 returnees (maximum *N* = 129)	Age 90 nonreturnees (maximum *N* = 421)
Age (years)	79.1 (0.6)	83.4 (0.5)		86.6 (0.4)		90.1 (0.1)	
Verbal Fluency age 79	40.0 (12.3)	41.4 (12.2)	38.1 (12.3)	42.3 (11.7)	38.3 (12.5)	42.9 (11.8)	39.1 (12.4)
Verbal Fluency age 83		39.8 (12.7)		40.9 (12.0)	36.9 (14.3)	42.1 (12.2)	38.6 (12.9)
Verbal Fluency age 87				40.0 (12.3)		42.3 (12.0)	36.3 (11.9)
Verbal Fluency age 90						39.5 (13.3)	
Logical Memory age 79 (/100)	31.6 (12.8)	33.7 (12.7)	28.8 (12.5)	34.5 (12.8)	29.5 (12.4)	35.3 (12.8)	30.5 (12.6)
Logical Memory age 83 (/100)		33.1 (14.3)		34.4 (14.2)	29.5 (14.2)	36.5 (13.6)	30.9 (14.4)
Logical Memory age 87 (/100)				32.9 (14.6)		36.4 (13.8)	27.3 (14.2)
Logical Memory age 90 (/100)						33.1 (16.7)	
Raven’s Matrices age 79	31.2 (8.8)	32.5 (8.4)	29.3 (9.0)	33.1 (8.4)	29.7 (8.9)	34.2 (8.3)	30.2 (8.8)
Raven’s Matrices age 83		29.7 (9.2)		30.8 (8.7)	27.0 (9.9)	32.4 (8.2)	28.0 (9.4)
Raven’s Matrices age 87				27.8 (9.2)		29.7 (8.7)	
Raven’s Matrices age 90						26.3 (8.7)	
Sex (% male)	41.8%						
Education (years)	10.9 (2.5)						
Social class (1–5)	2.2 (0.9)						
Alcohol (units per week)	5.5 (9.3)						
Smoking (% current/ex/never)	7.3%/49.4%/41.8%						

*Note:* Figures are given as mean (*SD*) unless otherwise specified. The mean age at each wave is used throughout to describe the testing occasions (i.e., ages 79, 83, 87, and 90). The covariates are given for age 79 (Wave 1) only as these were entered into the growth curve models. Cognitive ability data are shown for the fullest available sample at each wave.

### Retrospective Physical Activity

Three hundred and seventy-six individuals answered the items relating physical activity at 20–35, 40–55, and 60–75 years of age. In each age group, responses ranged from moving only in connection with necessary (household) chores (1) to keep-fit/heavy exercise or competitive sports several times a week (6). Although the modal response was walking or other outdoor activities several times per week (3), during each age period there were statistically significant decreases in the mean physical activity level with increasing age: at 20–35 years of age, the mean level was 3.3 (*SD* = 1.4), decreasing to 3.0 (*SD* = 1.2) at 40–55 years, and then to 2.6 (*SD* = 1.0) at 60–75 years [for the difference in mean physical activity level between 20–35 and 40–55 years of age, *t*(375) = 7.591 (*p* < .001) and between 40–55 and 60–75 years of age, *t*(374) = 7.505 (*p* < .001)].

Men reported being more physically active than women during each of the three age periods: the mean level of physical activity at 20–35 years of age was 3.8 (*SD* = 1.5) for men versus 3.0 (*SD* = 1.2) for women [*t*(291.7) = 5.432 (*p* < .001)]; at 40–55 years of age, the mean level was 3.1 (*SD* = 1.2) for men versus 2.8 (*SD* = 1.1) for women [*t*(374) = 2.757 (*p* = .006)]; and at 60–75 years of age, the mean level was 2.9 (*SD* = 1.0) for men versus 2.4 (*SD* = 0.9) for women [*t*(374) = 4.401 (*p* < .001)].

### Retrospective Leisure Activity

EFA were conducted separately for the three age periods. In each case, the overall measures of sampling adequacies were acceptable (ranging from .71 to .76); however, the item “going to pubs or social clubs” had the lowest individual value for each age period (.45–.47) and was therefore removed from the analyses. Examination of the scree plots from EFA of the remaining 14 activities for each age suggested the extraction of a single factor accounting for between 23.3% and 24.9% of the variance. Item loadings on these first unrotated factors are shown in Supplementary Table 1. The standardized residuals were used to define activity scores (referred to hereinafter as leisure activity 20–35, 40–55, and 60–75).

Correlations among the leisure activity scores are shown in Supplementary Table 2. During each period, women had higher leisure activity scores than men: at 20–35 years of age, women had a mean of 19.0 (*SD* = 5.5) versus 16.4 (*SD* = 5.2) for men [*t*(372) = −4.569 (*p* < .001)]; at 40–55 years of age, women had a mean of 17.6 (*SD* = 5.8) versus 15.7 (*SD* = 5.3) for men [*t*(371) = −3.359 (*p* = .001)]; at 60–75 years of age, women had a mean of 16.3 (*SD* = 5.5) versus 14.4 (*SD* = 5.3) for men [*t*(370) = −3.310 (*p* = .001)]. There were also statistically significant decreases in leisure activity with increasing age: at 20–35 years of age, the mean level was 17.9 (*SD* = 5.5), decreasing to 16.8 (*SD* = 5.7) at 40–55 years of age, and then to 15.6 (*SD* = 5.5) at 60–75 years of age [for the difference in mean leisure activity between 20–35 and 40–55 years of age, *t*(370) = 6.794 (*p* < .001) and between 40–55 and 60–75 years of age, *t*(369) = 7.570 (*p* < .001)].

### Age-79 Activity

At age 79, participants reported doing vigorous physical activity (for at least 20 minutes) on a mean of 6.2 days/month (*SD* = 8.5). Men were significantly more physically active, recording they did physical activity on an average of 7.7 days/month (*SD* = 9.1) compared with 5.1 days/month (*SD* = 7.9) for women [*t*(401.3) = 3.281 (*p* = .001)].

An EFA was conducted on the 13 leisure activity items at age 79, suggesting the extraction of one to three factors. For consistency with the retrospective data, the single factor solution accounting for 23.6% of the variance was retained. Factor loadings are given in Supplementary Table 3 and the standardized residual defined a leisure activity score at age 79.

### Modeling Cognitive Change

Before inclusion of the activity factors and covariates, measurement invariance across the waves of cognitive assessment was tested as described in the Statistical analysis section. From the most to least constrained model, there was no or minimal change in fit: CFI = .99, AIC = 8,088.98, Chi-square = 67.25 (df = 54, *p* = .106), and RMSEA = .021 (90% CI = 0.000–0.036) in the first model, compared with CFI = .99, AIC = 8,083.37, Chi-square = 74.05 (df = 60, *p* = .105), and RMSEA = .021 (90% CI = 0.000–0.035) in the final model. The final model suggested that there was significant cognitive decline over the four waves of follow-up (slope = −.039, *p* < .001) and that there was significant variance in this (.003, *p* < .001).

### Activity and Cognitive Change

In the unadjusted model, the only activity factor associated with intercept was leisure activity at 40–55 years of age (.333, *p* = .036), illustrated in Figure 1 with path coefficients for all activity factors in Table 2. Individuals who were more active in socio-intellectual type activities in midlife had a higher level of cognitive ability in later life. This positive association remained after inclusion of the covariates (.320, *p* = .030), with age-11 cognitive ability and education also being positively associated with intercept (.544, *p* < .001, and .171, *p* = .047, respectively). Table 2 displays the path coefficients for this adjusted model which accounted for 51.1% of the variance in intercept.

**Table 2.** Summary of Latent Growth Curve Model of Activity Predicting Cognitive Ability and Change From Age 79 to 90

	Unadjusted model		Adjusted model		Unadjusted model		Adjusted model	
	Intercept path coefficient (*p*)	Slope path coefficient (*p*)	Intercept path coefficient (*p*)	Slope path coefficient (*p*)	Intercept path coefficient (*p*)	Slope path coefficient (*p*)	Intercept path coefficient (*p*)	Slope path coefficient (*p*)
Leisure activity 20–35	−.188 (.215)	.142 (.455)	−.156 (.270)	.119 (.588)				
Leisure activity 40–55	**.333 (.036**)	.070 (.720)	**.320 (.030**)	.026 (.907)				
Leisure activity 60–75	.094 (.428)	−.139 (.340)	−.133 (.253)	−.167 (.306)				
Lifetime leisure activity					**.225 (.002**)	.076 (.485)	.048 (.538)	−.008 (.949)
Leisure activity 79	.019 (.785)	.049 (.655)	.087 (.211)	−.007 (.950)	.043 (.533)	.029 (.780)	.092 (.167)	−.038 (.737)
Physical activity 20–35	.097 (.331)	−.240 (.082)	.077 (.412)	−.171 (.250)				
Physical activity 40–55	−.039 (.731)	.039 (.809)	−.015 (.881)	.062 (.717)				
Physical activity 60–75	.073 (.486)	.181 (.168)	−.007 (.944)	**.265 (.045**)				
Lifetime physical activity					.110 (.153)	−.047 (.670)	.053 (.454)	.099 (.328)
Physical activity 79	−.040 (.588)	−.102 (.287)	−.032 (.631)	−.097 (.323)	−.038 (.607)	−.092 (.346)	−.049 (.465)	−.082 (.411)
Sex			−.096 (.263)	.019 (.066)			−.119 (.176)	**.020 (.035**)
Age-11 cognitive ability			**.544 (<.001**)	.048 (.619)			**.535 (<.001**)	.074 (.454)
Education			**.171 (.047**)	−.021 (.842)			.165 (.057)	−.017 (.872)
Social class			−.048 (.550)	.103 (.361)			−.043 (.585)	.135 (.233)
Smoking			.068 (.372)	−.040 (.706)			.046 (.536)	−.052 (.624)
Alcohol			.069 (.283)	−.179 (.081)			.065 (.310)	−.159 (.160)

*Note:* The figures represent standardized path coefficients from the listed variable to the intercept and slope parameters (Figure 1). The latent general cognitive ability factors were defined by three cognitive tests on each occasion. Leisure and physical activity 20–35, 40–55, and 60–75 were collected by retrospective self-report during the age-83 assessment (Wave 2); leisure and physical activity 79 were assessed at the age-79 assessment (Wave 1). Lifetime leisure/physical activity represents cumulative scores from the age 20–35, 40–55, and 60–75 assessments. The unadjusted model included only the activity factors, whereas the adjusted model additionally included sex, age-11 cognitive ability, education, social class, smoking, and alcohol. Significant paths are highlighted in bold.

In terms of slope, there were no associations between activity (physical or leisure) and cognitive change in the unadjusted model (though the slope parameter remained significant in this model including the activity factors (−.034, *p* < .001), and there was significant variance in this (.002, *p* < .001)). However, after inclusion of the covariates, the association between physical activity at 60–75 years of age and slope became significant (from .181, *p* = .168, in the unadjusted model, to .265, *p* = .045). That is, after accounting for a range of relevant covariates, individuals who were more physically active in later life experienced less cognitive decline over the 11 years of follow-up from age 79 to 90. In the fully adjusted model, the slope parameter was significant (−.046, *p* < .001) and there was significant variance in this (.002, *p* < .001). The adjusted model accounted for 19.2% of the variance in slope.

In models including only the covariates, 44.4% of the variance in intercept was accounted for and 5.9% of the variance in slope. The activity factors therefore accounted for about 6.7% of the variance in intercept and 12.5% in slope.

To examine whether the associations with specific age periods reflected one score accounting for a more cumulative effect of activity, final growth curve models replaced the leisure and physical activity scores at ages 20–35, 40–55, and 60–75 years with lifetime leisure and lifetime physical activity scores (a summed score of the three age periods for physical activity and the standardized residual from an EFA of the 14 activity items from all three age periods simultaneously for leisure activity), summarized in Table 2. In the unadjusted model, lifetime leisure activity was associated with intercept (.225, *p* = .002); in the model including the covariates, this association was reduced (.048, *p* = .538). None of the activity scores were associated with slope (though the significant decline and variance in slope remained unchanged from earlier models). Childhood cognitive ability was associated with level .535 (*p* < .001), whereas sex was associated with slope (.020, *p* = .035), the men showing greater decline.

## Discussion

Retrospective lifestyle assessments may add value to existing aging cohorts. In the LBC1921, there was some evidence of sensitive periods for activity participation in terms of the associations observed with cognitive ability and change. Accounting for demographic covariates and, importantly, childhood cognitive ability, engagement in activities of a social or intellectual nature in midlife was associated with higher cognitive ability level, whereas physical activity in later life was associated with less cognitive decline within old age. Findings from a study of middle-aged adults that was also able to account for childhood cognitive ability produced a similar pattern of results (Richards et al., 2003). It has been suggested that leisure time activities that are socially or intellectually engaging might be more important for cognitive development across midlife by increasing or maintaining cognitive ability level, rather than protecting against cognitive decline per se (Bielak et al., 2012).

The lack of associations between the either of the cumulative lifetime activity scores and cognitive ability and change might be taken to imply there are specific periods in which engagement in socio-intellectual or physical activities might be beneficial. It is important to note, however, while this account would be consistent with the current findings, others have shown associations with cumulative measures (Wilson, Barnes, et al., 2003), albeit not adjusting for the stability of cognitively ability across the lifecourse. The suggestion that leisure and physical activities might offer benefits during different time periods is therefore offered tentatively, especially given cognitive ability was not assessed across the same lifecourse periods. Those large midlife cohorts now approaching old age will allow this to be more fully addressed. Interestingly, neither activity measure from age 79 was associated with cognitive ability level or change in the context of the retrospective activity measures. The findings therefore support the need to take a lifecourse approach in identifying the determinants of cognitive aging (Andel et al., 2015; Curtis, Huxhold, & Windsor, in press), and indeed that strategies to enhance cognitive ability or protect against cognitive decline may need to begin before old age.

In assessing retrospective physical activity and cognitive change, Dik, Deeg, Visser, and Jonker (2003) highlighted potential sex differences, such that only men benefitted from higher physical activity in early life. Given the sample size, the current findings were not explored separately by sex (introducing interaction terms resulted in model convergence issues), though differences in the type and timing of activity required for later cognitive benefits would be of interest. Particularly in older cohorts such as the LBC1921, the life experiences of men and women are likely to have been quite different; women of this generation would have been afforded fewer educational and occupational opportunities and would have been less likely to work outside the home, for example. 

If there is a cognitive benefit from higher physical activity participation, the pathways underlying the association could be numerous, but mechanisms via a reduction in cardiovascular risk factors or disease are commonly cited (Hendrie et al., 2006; Yaffe, Barnes, Nevitt, Lui, & Covinsky, 2001), as are accounts of the potential neurogenic and angiogenic effects (Hertzog et al., 2009).

In terms of leisure activity, midlife participation appeared to predict a higher level of cognitive ability in old age, independent of the level of prior ability, but not changes in cognitive ability in later life. Such findings are relevant to the differential preservation versus preserved differentiation debate as they suggest that reported associations between increased activity and better cognitive outcomes are not entirely due to individuals of higher initial ability being more active. The findings therefore complement and extend those from a related cohort in which the positive association between leisure activity and cognitive ability at age 70 was attenuated after accounting for the lifetime stability of cognitive ability (Gow et al., 2012). In both cohorts, there were therefore no reported associations between later life leisure activity participation and cognitive ability. This is contrary to much of the literature where such associations are commonly reported (Baer et al., 2013; Lee et al., 2014), though these studies rarely address the differential preservation versus preserved differentiation issue. Although the current results support the existence of a positive association between activity and cognitive ability independently of prior ability, it might be suggested that when data are only available in later life the reported associations may be an artifact of a benefit derived from activity participation earlier in the life span. The latter suggestion needs to be further explored, in data sets with more robust lifetime activity data than would be possible by retrospective methods.

Lifetime activity involving a mental component was predictive of cognitive ability according to Wilson and colleagues (2003). Due to the nature of the current analysis, there was no specification between social and intellectual pastimes in the three age periods. This was not pursued as separate domain scores would each have been described by a small number of items, though the cognitive stimulation resulting from increased activity is often suggested as the key to any observed protective effect (Hertzog et al., 2009). Use or disuse of mental abilities (via engagement in activities) may therefore lead to actual structural or functional changes in the brain (number of synapses, speed of dendrites, etc.) which will then determine an individual’s future engagement with their environment (Anstey & Christensen, 2000; Kramer et al., 2004).

These changes in the central nervous system are often linked to the notion of “reserve capacity,” that is “the amount of damage that can be sustained before reaching a threshold for clinical expression” (Stern, 2002, p. 449). Although the nature of this reserve is often unspecified, the current findings suggest that this reserve might be better conceived as simply the level of functioning an individual achieves across midlife (given that longitudinal studies have highlighted the potential for continued development of certain cognitive abilities through the 30s to 50s) (Hedden & Gabrieli, 2004; Schaie, 2013). That is, consider two individuals equal in all regards on entering midlife. From the current findings, the individual engaging in more socio-intellectual activities throughout midlife would be predicted to experience a greater increase (or reduced decline) in their cognitive abilities relative to their less active peer. The difference in cognitive ability level of the active versus inactive individual on entering old age might therefore be considered the “reserve capacity” associated with midlife activity. If the two individuals experienced the same rate of cognitive decline from that point forward, the benefit of entering old age at a higher level of cognitive ability (greater reserve capacity) would be that the active individual takes longer to reach any impairment threshold.

### Strengths and Limitations

Although data collected contemporaneously in studies spanning years or decades of follow-up would be preferred (Andel et al., 2015), there are also advantages in utilizing retrospective methods (Dik et al., 2003; Fritsch et al., 2005). Utilizing retrospective assessment can increase the time over which individuals are assessed. In the current study, these methods were employed to collect data between the childhood assessments and the initiation of the aging study in later life. Retrospective assessment will, however, always be prone to issues of recall bias and questions of reliability. Those concerns over recall bias are perhaps particularly relevant to studies with older adults given age-related declines in memory skills (Hertzog et al., 2009). Retrospective methods have been increasingly used in older cohorts (e.g., Wilson & colleagues, 2003), though they must be interpreted with particular caution given the likely collinearity issues in asking participants to complete the same items while reflecting over an extended period of time. In the current study, we reported the cumulative lifetime scores to reduce such concerns (modeling constraints meant it was not possible to employ growth curve procedures to explore changes in leisure and physical activities at the same time as cognitive ability, which would have been the more appropriate method for addressing potential collinearity). Using cumulative lifetime activity measures does not, of course, diminish the recall bias issue, but perhaps more accurately reflects a lifetime exposure at the most general level. In those final models, no activity and cognitive ability/change associations were reported and so as above, our findings are interpreted tentatively and offered for replication.

Bearing in mind such caveats, it may be that the use of retrospective assessments can add value, especially when aging cohorts have detailed phenotypic information across later life. That said, the age periods used currently were relatively large and do not address the many likely within-individual differences in activity (and other unmeasured factors) that will have occurred during each 15-year period; our intention was, however, to broadly capture between-individual variation in general activity participation patterns across the lifecourse. We were not seeking, nor did we think it plausible, that participants would pinpoint exact activity levels. Rather they were instructed to reflect on the time period and answer generally. In any given age period, there are likely many factors that the participants will have encountered (marriage, starting a family, unemployment, etc.). Some of these may be more common in one age period than others, but we were not able to assess all such factors. Though cognitive abilities were not assessed during the retrospective age periods selected, they might, very roughly, map onto the period where peaks in processing speed would be seen in the younger adulthood period followed by decline, stability of most abilities across midlife, and general declines in later adulthood (Hedden & Gabrieli, 2004).

Of general concern in any study, including the current one, is that of selective attrition (in those who returned for cognitive follow-ups and who chose to complete the retrospective assessments). In general, the cohort will have suffered restriction of range over time. The associations are likely underestimates though no selectivity analyses were performed.

The LBC1921 benefits from the presence of childhood cognitive ability data, allowing the differential preservation versus preserved differentiation question to be considered. Although a battery of tests were employed in later life, these were currently only considered as defining a general cognitive ability factor. Analyzing cognitive tests separately to explore potential domain-specific associations is not advised, although it is an approach common in the cognitive aging literature. To consider specific domains of cognitive ability (e.g, processing speed, reasoning, or memory), multiple markers of each would be required. The activity measures used, particularly for physical activity, were relatively crude (though comparable with the previous research from where they were drawn). It would of course be advantageous to consider other aspects of physical activity, such as the type, intensity, and duration, or some index of energy expenditure. This could more precisely indicate the level of physical activity required to produce a beneficial effect on cognition. The retrospective assessment of activity participation had to remain brief, however, as participants were required to reflect on three different periods in their distant past.

Assessing a greater number of activities would have allowed specific aspects of engagement (such as social versus intellectual) to be considered rather than simply overall activity. The analyses did not support extraction of different activity factors from the retrospective assessment. Although differentiating these types of activities would be of interest, it is perhaps an advantage to consider activity broadly in the current analyses given concerns over recall bias. Although it is not possible to address such limitations in the LBC1921, retrospective assessments have recently been completed in a related cohort, currently undergoing cognitive assessments at age 79 (with data available from childhood and at ages 70, 73, and 76 years). When data collection is complete, it will be possible to replicate the current results, in addition to exploring differences across cognitive ability and leisure activity domains, and by sex. Replication is particularly important as a lack of power may have failed to identify slope determinants with small effects. Furthermore, the number of paths included increased the possibility of type 1 errors.

### Conclusions

Given activity participation may be “more directly amenable to psychological intervention” (Schaie, 1984, p. 476) than other influences on cognitive aging, the suggestion that participation in leisure or physical activities in mid or later life might be beneficial is important. In the LBC1921, greater leisure activity appeared to predict the level of ability in old age, whereas physical activity was associated with the maintenance of this over time. Engagement in a range of activities across the lifecourse may be necessary to ensure cognitive vitality in later life although replication of these effects is clearly required before intervention strategies would be suggested.

## Supplementary Material

Please visit the article online at http://psychsocgerontology.oxfordjournals.org/ to view supplementary material.

## Funding

This work was supported by the Biotechnology and Biological Sciences Research Council (15/SAG09977 to I. J. Deary); a Royal Society Wolfson Research Merit Award (to I. J. Deary); the Chief Scientist Office of the Scottish Government (CZG/3/2/79 to I. J. Deary; CZB/4/505 to I. J. Deary and A. J. Gow; and ETM/55 to I. J. Deary and A. J. Gow); the University of Edinburgh Development Trust Research Fund (to I. J. Deary); and a Royal Society of Edinburgh/Lloyds TSB Foundation for Scotland Studentship (to A. J. Gow). This work was undertaken by the University of Edinburgh Centre for Cognitive Ageing and Cognitive Epidemiology, part of the cross council Lifelong Health and Wellbeing initiative. Funding from the Biotechnology and Biological Sciences Research Council and Medical Research Council is gratefully acknowledged (MR/K026992/1).

## Conflict of Interest

The authors report no conflicts of interest.

## Supplementary Material

Supplementary Data
