# Supplementary material for: Lifecourse Activity Participation From Early, Mid, and Later Adulthood as Determinants of Cognitive Aging: The Lothian Birth Cohort 1921
Source: J Gerontol B Psychol Sci Soc Sci. 2016 Oct 7;72(1):25–37. doi: 10.1093/geronb/gbw124 (PMC5156497; doi:10.1093/geronb/gbw124)
Supplement: Supplementary Data [file supp_72_1_25__index.html]

Lifecourse Activity Participation From Early, Mid, and Later Adulthood as Determinants of Cognitive Aging: The Lothian Birth Cohort 1921 — Lifecourse Activity Participation From Early, Mid, and Later Adulthood as Determinants of Cognitive Aging: The Lothian Birth Cohort 1921 — Supplementary Data 

# Lifecourse Activity Participation From Early, Mid, and Later Adulthood as Determinants of Cognitive Aging: The Lothian Birth Cohort 1921

## Supplementary Data

Data files

- Supplementary Data - Supplementary Data
