# Supplementary material for: Lifecourse Activity Participation From Early, Mid, and Later Adulthood as Determinants of Cognitive Aging: The Lothian Birth Cohort 1921
Source: J Gerontol B Psychol Sci Soc Sci. 2016 Oct 7;72(1):25–37. doi: 10.1093/geronb/gbw124 (PMC5156497; doi:10.1093/geronb/gbw124)
Supplement: Supplementary Data [file supp_gbw124_Supplementary_Tables_1_4.docx]

Supplementary Table 1

Retrospective leisure activity factor loadings

|  | Age 20-35 | Age 40-55 | Age 60-75 |
| --- | --- | --- | --- |
| Trips to the theatre, galleries or museums | **.60** | **.66** | **.72** |
| Visits to the library | **.60** | **.53** | **.49** |
| Writing | **.50** | **.59** | **.47** |
| Going to the cinema or restaurants | **.49** | **.53** | **.56** |
| Reading a book | **.49** | **.43** | **.36** |
| Going to sporting events or concerts | **.47** | **.55** | **.60** |
| Participation in social groups | **.44** | **.48** | **.35** |
| Visits to friends or family | **.41** | **.39** | **.46** |
| Church or religious activities | **.35** | **.33** | **.33** |
| Listening to the radio | **.35** | **.36** | **.32** |
| Reading a newspaper or magazine | **.32** | **.34** | .18 |
| Playing games (like cards, chess, bingo or crosswords) | .25 | **.31** | .19 |
| Study course at work or evening classes | .24 | .24 | .34 |
| Watching television | .11 | .13 | -.01 |

Note. EFA were conducted on the 14 leisure activities separately for each age period. Loadings over 0.3 are highlighted in bold. Standardised residuals from the EFA defined leisure activity scores at ages 20-35, 40-55 and 60-75.

Supplementary Table 2

Correlations between leisure and physical activity scores derived from latent growth curve model

|  | Physical activity 79 | Physical activity 20-35 | Physical activity 40-55 | Physical activity 60-75 | Leisure activity 79 | Leisure activity 20-35 | Leisure activity 40-55 |
| --- | --- | --- | --- | --- | --- | --- | --- |
| Physical activity 79 |  |  |  |  |  |  |  |
| Physical activity 20-35 | 0.208 |  |  |  |  |  |  |
| Physical activity 40-55 | 0.248 | 0.720 |  |  |  |  |  |
| Physical activity 60-75 | 0.291 | 0.514 | 0.677 |  |  |  |  |
| Leisure activity 79 | 0.139 | 0.160 | 0.158 | 0.127 |  |  |  |
| Leisure activity 20-35 | 0.104 | 0.224 | 0.189 | 0.136 | 0.240 |  |  |
| Leisure activity 40-55 | 0.108 | 0.214 | 0.199 | 0.189 | 0.35 | 0.829 |  |
| Leisure activity 60-75 | 0.155 | 0.170 | 0.160 | 0.204 | 0.399 | 0.678 | 0.779 |

Note. Leisure and physical activity 20-35, 40-55 and 60-75 were collected by retrospective self-report during the age-83 assessment (Wave 2); leisure and physical activity 79 were assessed at the age-79 assessment (Wave 1). Correlations were derived from the growth curve model including all cognitive ability measures, covariates and activity variables.

Supplementary Table 3

Age-79 leisure activity factor loadings

|  | Loading |
| --- | --- |
| Visiting friends | **.76** |
| Talking to friends | **.66** |
| Visiting relatives | **.59** |
| Talking to relatives | **.48** |
| Day or overnight trips | **.46** |
| Visits to cinema, sporting events, restaurants | **.46** |
| Attending church | **.34** |
| Participation in social groups | **.32** |
| Unpaid community work | .25 |
| Listening to the radio | .21 |
| Learning a language | .14 |
| Playing cards, games, bingo | .11 |
| Paid community work | .02 |

Note. Loadings over 0.3 are highlighted in bold. Standardised residuals from the EFA defined a leisure activity score at age 79.

Supplementary Table 4

Correlations between cognitive ability tests derived from latent growth curve model

|  | VF79 | LM79 | RSPM79 | VF83 | LM83 | RSPM83 | VF87 | LM87 | RSPM87 | VF90 | LM90 |
| --- | --- | --- | --- | --- | --- | --- | --- | --- | --- | --- | --- |
| VF79 |  |  |  |  |  |  |  |  |  |  |  |
| LM79 | 0.154 |  |  |  |  |  |  |  |  |  |  |
| RSPM79 | 0.221 | 0.345 |  |  |  |  |  |  |  |  |  |
| VF83 | 0.768 | 0.219 | 0.254 |  |  |  |  |  |  |  |  |
| LM83 | 0.156 | 0.773 | 0.323 | 0.250 |  |  |  |  |  |  |  |
| RSPM83 | 0.226 | 0.329 | 0.786 | 0.295 | 0.320 |  |  |  |  |  |  |
| VF87 | 0.735 | 0.134 | 0.252 | 0.824 | 0.182 | 0.326 |  |  |  |  |  |
| LM87 | 0.1568 | 0.665 | 0.347 | 0.237 | 0.754 | 0.397 | 0.269 |  |  |  |  |
| RSPM87 | 0.144 | 0.263 | 0.759 | 0.270 | 0.327 | 0.811 | 0.296 | 0.430 |  |  |  |
| VF90 | 0.711 | 0.130 | 0.290 | 0.807 | 0.220 | 0.347 | 0.812 | 0.281 | 0.277 |  |  |
| LM90 | 0.102 | 0.574 | 0.257 | 0.214 | 0.651 | 0.396 | 0.179 | 0.765 | 0.372 | 0.250 |  |
| RSPM90 | 0.167 | 0.240 | 0.7029 | 0.287 | 0.3219 | 0.713 | 0.293 | 0.424 | 0.723 | 0.387 | 0.459 |

Note. VF = Verbal Fluency, LM = Logical Memory, and RSPM = Raven’s Standard Progressive Matrices. Correlations were derived from the growth curve model including all cognitive ability measures, covariates and activity variables.
